# Unexpected association between subclinical hearing loss and restorative sleep in a middle-aged and elderly Japanese population

**DOI:** 10.1186/s13104-018-3315-8

**Published:** 2018-03-27

**Authors:** Kei Nakajima, Eiichiro Kanda, Kaname Suwa

**Affiliations:** 1 School of Nutrition and Dietetics, Faculty of Health and Social Services, Kanagawa University of Human Services, 1-10-1 Heisei-cho, Yokosuka, Kanagawa 238-8522 Japan; 2 Department of Nephrology, Tokyo Kyosai Hospital, Meguro, Tokyo Japan; 3 Saitama Health Promotion Corporation, 410-1 Yoshimicho, Hikigun, Saitama 355-0133 Japan

**Keywords:** Hearing loss, High frequency, Bilateral hearing loss, Restorative sleep, Cardiometabolic risk, Age-related hearing loss, Cognitive impairment

## Abstract

**Objective:**

Hearing loss may be associated with certain sleep abnormalities. We recently reported that subclinical hearing loss (SHL) was more prevalent in individuals in a broad Japanese population who slept longer than 8 h; however, the underlying mechanism was unknown. Therefore, we investigated the association between SHL and self-reported restorative sleep (RS), assessed by questionnaire, in a database of 33,888 Japanese aged 40–69 years without overt or diagnosed hearing loss (20,225 men, 13,663 women).

**Results:**

The proportion of individuals with RS (more than half of the subjects) was significantly higher in the group with bilateral than with unilateral SHL at 4000 Hz and intact hearing; however, that was not the case at 1000 Hz, independent of age (*P* < 0.0001, two-way analysis of variance). Multivariate logistic regression analysis showed that bilateral SHL at 4000 Hz, but not at 1000 Hz, was significantly associated with RS. This relationship was independent of potential relevant confounders, including age, sex, and cardiometabolic risk factors. The present study extends our earlier work by revealing an unexpected association between early hearing impairment and satisfactory sleep in a middle-aged and elderly population. This association requires further confirmation regarding the possible underlying mechanism and clinical relevance.

**Electronic supplementary material:**

The online version of this article (10.1186/s13104-018-3315-8) contains supplementary material, which is available to authorized users.

## Introduction

Hearing loss is one of the most important disabilities in hampering social interaction and quality of life [1–5]. The degree of hearing impairment usually advances with age, but significant variation occurs among individuals [1, 6, 7]. Subclinical hearing loss (SHL) is mostly asymptomatic and underappreciated in the general population [2, 5]; thus, it tends to advance gradually, even in middle-aged people [8, 9].

Good sleep is critical for general health throughout life; however, both short and long sleep duration are associated with cardiometabolic morbidities, such as obesity and type 2 diabetes [10–12]. In a previous study of a medical database containing details of a broad Japanese population who underwent a health check-up in 2007 or 2008 [13], we found, surprisingly, that SHL at 4000 Hz was more prevalent in individuals who slept longer (≥ 8 h). In the present study, we investigated the association between SHL and self-reported restorative sleep (RS) in a different database of middle-aged and elderly Japanese people.

## Main text

### Methods

This cross-sectional study utilized data recorded during medical check-ups of people living or working in Saitama, a suburb of Tokyo, Japan [14]. We reviewed the data for 62,459 individuals who underwent a health check-up at the Saitama Health Promotion Corporation in 2012. We excluded from the study 277 subjects with overt or diagnosed hearing loss (including those with ongoing treatment or past history). Older subjects aged 70 years or more (n = 677) were excluded from the main analysis: the quality of sleep can be influenced by various age-related conditions, such as cognitive and urinary tract disease [15, 16]. We also excluded younger subjects aged 20–39 years (n = 27,617) because the prevalence of SHL is very low in the young generation. After these exclusions, 33,888 apparently healthy subjects aged 40–69 years with complete data were enrolled. However, in a subsequent sub-analysis, we removed the restriction for age and conducted the same analysis (Additional file 3: Figure S1) because observed SHL might be classified into noise or other-induced hearing loss rather than age-related hearing loss (ARHL).

All anthropometric and laboratory tests were conducted in the early morning after overnight fasting. Serum parameters were measured using standard methods on Hitachi autoanalyzers (Tokyo, Japan) at the Saitama Health Promotion Corporation. HbA1c (Japan Diabetes Society [JDS]) was converted to HbA1c (National Glycohemoglobin Standardization Program [NGSP]) units using the officially certified formula: HbA1c (NGSP) (%) = 1.02 × JDS (%) + 0.25% [17]. A hearing test with a headphone was individually conducted in a restricted quiet room or place by trained staff using an ordinary audiometer. Following several tests for decisions at each frequency, SHL was defined as a pure-tone average hearing loss of > 25 dB at high (4000 Hz) and low (1000 Hz) frequencies.

As in other studies [18, 19], RS was determined based on a positive response to the question “Do you get sufficient rest from sleep?” That was included in a questionnaire developed by Japan’s Ministry of Health, Labour and Welfare in 2008 for detecting unhealthy lifestyles [20]. Subjects were primarily divided into three groups according to the absence or presence of SHL at 4000 Hz or 1000 Hz, unilaterally or bilaterally: intact hearing, unilateral SHL, and bilateral SHL.

### Statistical analysis

Data are expressed as means ± standard deviations. We evaluated differences in continuous and categorical variables among the three SHL groups using one-way analysis of variance (ANOVA) and χ^2^ tests, respectively. We conducted post hoc analysis using the Bonferroni test and an additional χ^2^ test (*P* < 0.013) between two specific groups. RS was investigated using two-way ANOVA with age-group (every 10 years) as one factor and SHL condition as another. We used multivariate logistic regression models to evaluate associations between RS and SHL with or without adjustment for relevant confounders, including various cardiometabolic risk factors. Logistic regression models yielded odds ratios (ORs) and 95% confidence intervals (95% CIs). To investigate the combined effect of bilateral SHL at 4000 and 1000 Hz on RS in the logistic regression analysis, in addition to the above three groups, we divided subjects into four groups according to the presence or absence of bilateral SHL at 4000 and 1000 Hz: not bilateral at either frequency; bilateral at 1000 Hz but not at 4000 Hz; not bilateral at 1000 Hz but bilateral at 4000 Hz; and bilateral at both frequencies. We also conducted separate analyses of men and women: in our previous study, we found that unlike women, men showed a significant association with hearing loss at 4000 Hz [13]. We performed statistical analyses using Statview, version 5.0 (SAS Institute; Cary, NC, USA). *P* < 0.05 was considered significant.

## Results

**Fig. 1:**
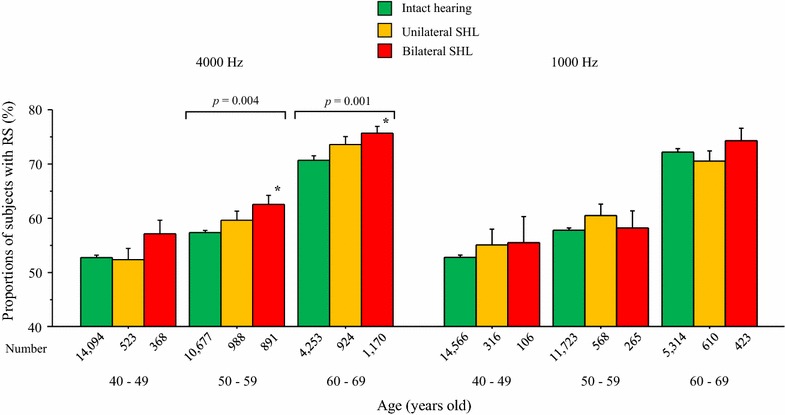
Proportions of subjects with RS by age-group The small vertical bars represent the standard error with RS numbered as 1 and non-RS as 0. The proportion of subjects with RS in their 50 s and 60 s significantly rose with increasing SHL (*P* = 0.004 and *P* = 0.001, respectively, one-way ANOVA). *Significant difference compared with intact hearing (Bonferroni test) in each age-group. *RS* restorative sleep

The subjects’ characteristics appear in Table 1. Individuals with unilateral or bilateral SHL at 4000 Hz were older than those with intact hearing and were more likely to be male. Most continuous variables and categorical parameters rose significantly with increasing SHL (all *P* < 0.0001, ANOVA). As shown in Fig. 1, the proportion of individuals with RS (which was more than half of subjects) was significantly higher in the group with bilateral than with unilateral SHL at 4000 Hz and intact hearing, but not at 1000 Hz (*P* < 0.0001 generally, two-way ANOVA). When the analysis was conducted for each age-group, a significant increase in RS was evident in subjects in their 50s and 60s (*P* = 0.004 and *P* = 0.001, respectively, one-way ANOVA). To reduce the effect of age, we categorized it every 5 years and conducted the same analysis: the results were similar, i.e., higher proportions of RS in bilateral SHL at 4000 Hz independent of age (*P* = 0.002 generally, one-way ANOVA, Additional file 1: Figure S1). We observed a significant rise in RS with SHL only in subjects aged 60–64 years (*P* = 0.01, one-way ANOVA). Notably, differences in the averages for age among the three categories in each group were all less than 1 year—even though age rose with increasing SHL in each age-group (all *P* < 0.0001, ANOVA, Additional file 2: Figure S1).

**Table 1:** Characteristics of subjects classified according to presence of SHL at 4000 Hz

Characteristic	Intact hearing	Unilateral SHL	Bilateral SHL
N (%)	29,024 (85.6)	2435 (7.2)	2429 (7.2)
Male, n (%)	15,911 (54.8)	2068 (84.9)	2246 (92.5)
Age (years)	50.7 ± 7.4	56.0 ± 7.4	58.0 ± 7.2
BMI (kg/m^2^)	23.4 ± 3.6	23.9 ± 3.2	23.7 ± 3.4
Systolic blood pressure (mmHg)	125 ± 17.1	131 ± 16.9	132 ± 17.8
Serum triglyceride (mg/dl)	98 (67–149)	113 (79–174)	111 (76–167)
Serum HDL-cholesterol (mg/dl)	60.2 ± 15.3	56.6 ± 15.0	55.3 ± 14.5
HbA1c (NGSP, %)	5.66 ± 0.7	5.82 ± 0.8	5.87 ± 0.9
Pharmacotherapy for			
Hypertension, n (%)	4037 (13.9)	546 (22.4)	616 (25.4)
Diabetes, n (%)	1031 (3.6)	171 (7.0)	202 (8.3)
Dyslipidemia, n (%)	2220 (7.6)	288 (11.8)	232 (9.6)
Current smokers, n (%)	7631 (26.3)	1009 (41.4)	1129 (46.5)
Everyday alcohol consumers, n (%)	7765 (26.8)	958 (39.3)	1127 (46.4)
Regular exercisers, n (%)^a^	7018 (24.2)	682 (28.0)	666 (27.4)
Past history of			
CVD, n (%)	711 (2.5)	118 (4.8)	125 (5.1)
Stroke, n (%)	321 (1.1)	63 (2.6)	57 (2.3)

Data are presented as mean ± SD, median (interquartile range), or numbers (%)

All *P* values determined by ANOVA and χ^2^ test for continuous and categorical variables, respectively, were < 0.0001. Differences between unilateral and bilateral SHL groups with regard to all continuous and categorical variables between two groups were statistically significant in post hoc Bonferroni testing and an additional χ^2^ test except for BMI, serum triglyceride, pharmacotherapy for hypertension, regular exercisers, and past history of CVD and stroke

Serum triglyceride concentrations were log-transformed before parametric analysis

*BMI* body mass index; *HDL* high-density lipoprotein; *CVD* cardiovascular disease; *SHL* subclinical hearing loss

^a^Regular exercise was defined as ≥ 30 min exercise per session at least twice a week

Logistic regression analysis showed that compared with intact hearing, bilateral SHL at 4000 Hz was significantly associated with RS: subjects with bilateral SHL at 4000 Hz were more likely to have RS. That finding remained significant even after full adjustment for confounding factors, including age, sex, smoking, alcohol consumption, current pharmacotherapy, and past history of cardiovascular disease and stroke (Model 3, Table 2). However, when subjects were categorized by sex, the statistical significance disappeared in women (Model 3, *P* = 0.09). The association between bilateral SHL at 1000 Hz and RS was not significant after adjustment for age and sex. Among confounding factors, habitual regular exercise and daily alcohol consumption were significantly associated with RS: ORs (95% CIs), 1.70 (1.61–1.79) and 1.25 (1.17–1.32), both *P* < 0.0001; however, past history of cardiovascular disease and current smoking were inversely associated with RS: 0.81 (0.70–0.92), *P* = 0.002 and 0.88 (0.83–0.93), *P* < 0.0001.

**Table 2:** Odds ratio (95% confidence interval) of SHL for RS

SHL at 4000 Hz	Both intact	Unilateral	Bilateral	
SSRS, n (%)	16,544 (57.0)	1542 (63.3)	1652 (68.0)	
Model 1	1 (reference)	1.30 (1.20–1.42)^***^	1.60 (1.47–1.75)^***^	
Model 2	1 (reference)	1.05 (0.96–1.15)	1.21 (1.10–1.32)^***^	
Model 3	1 (reference)	1.06 (0.97–1.16)	1.22 (1.11–1.34)^***^	
Model 3				
Men	1 (reference)	1.03 (0.94–1.14)	1.18 (1.10–1.32)^**^	
Women	1 (reference)	1.14 (0.92–1.42)	1.32 (0.96–1.81)	

SHL at 1000 Hz	Both intact	Unilateral	Bilateral	
N (% of total)	31,603 (93.3)	1494 (4.4)	791 (2.3)	
SSRS, n (%)	18,266 (57.8)	947 (63.4)	525 (66.4)	
Model 1	1 (reference)	1.26 (1.14–1.41)^***^	1.44 (1.24–1.67)^***^	
Model 2	1 (reference)	1.04 (0.93–1.16)	1.09 (0.94–1.27)	
Model 3	1 (reference)	1.04 (0.91.17)	1.10 (0.94–1.28)	

SHL at 4000 Hz	Not bilateral	Not bilateral	Bilateral	Bilateral
SHL at 1000 Hz	Not bilateral	Bilateral	Not bilateral	Bilateral
N (% of total)	31,019 (91.5)	440 (1.3)	2078 (6.1)	351 (1.0)
SSRS, n (%)	17,810 (57.4)	276 (62.7)	1403 (67.5)	249 (70.9)
Model 1	1 (reference)	1.25 (1.03–1.52)^*^	1.54 (1.40–1.69)^***^	1.81 (1.44–2.28)^***^
Model 2	1 (reference)	0.99 (0.81–1.21)	1.18 (1.07–1.30)^**^	1.29 (1.02–1.62)^*^
Model 3	1 (reference)	1.00 (0.82–1.22)	1.20 (1.08–1.32)^**^	1.30 (1.03–1.65)^*^
Model 3				
Men	1 (reference)	1.06 (0.80–1.41)	1.18 (1.06–1.31)^**^	1.18 (0.91–1.53)
Women	1 (reference)	0.93 (0.70–1.24)	1.10 (0.76–1.61)	1.91 (1.05–3.46)^*^

Model 1: unadjusted

Model 2: adjustment for age and sex

Model 3: Model 2 plus adjustment for current smoking (versus non-smoking), daily alcohol consumption (versus infrequent/no alcohol consumption), regular exercise (versus no regular exercise), pharmacotherapy (for hypertension, diabetes, or dyslipidemia), BMI, systolic blood pressure, triglyceride, HDL cholesterol, HbA1c (as continuous variables) and past history of CVD or stroke (versus no history)

*BMI* body mass index; *HDL* high-density lipoprotein; *CVD* cardiovascular disease; *SHL* subclinical hearing loss; *RS* restorative sleep

^*^ *P *< 0.05, ^**^ *P *< 0.01, ^***^ *P *< 0.0001

We categorized the subjects into four groups according to bilateral SHL at 4000 Hz and 1000 Hz: bilateral SHL at 4000 HL (but not bilateral SHL at 1000 Hz) and bilateral SHL at both frequencies were significantly associated with RS—even after full adjustment for confounders. After categorization by sex, the former association remained significant in men, and the latter association remained significant in women.

In a sub-analysis, we removed the age restriction for subjects and conducted the same analysis for SHL at 4000 Hz (n = 62, 182 in total, Additional file 3: Figure S1). Except for the oldest group, aged 70–79 years, the proportion of RS was highest in subjects with bilateral SHL—even with the youngest group, aged 20–29 years; however, no statistical difference was observed. In the logistic regression analysis for the whole study, compared with intact hearing, unilateral and bilateral SHL at 4000 Hz were significantly associated with RS—even after the same full adjustments for confounding factors: 1.20 (1.11–1.30) and 1.50 (1.38–1.64), respectively, both *P* < 0.0001 (data not shown).

## Discussion

This study determined that bilateral SHL (defined as > 25 dB at 4000 Hz), which may reflect hearing threshold elevation in aging, was significantly associated with RS, independent of relevant confounders, including age, sex, and cardiometabolic risk factors. In contrast to our previous study, the database used for the present study lacked data on sleep duration, [13]; thus, we could not confirm our previous findings using the same analysis. In the current study, RS was subjectively determined and was not validated by any confirmatory investigations; thus, this study should be considered preliminary in character. However, Matsumoto et al. [19] found non-RS to be significantly associated with shorter sleep duration, sleepiness, and frequent stress; subjects with RS had longer sleep duration, adequate sleep, and rare stress. Further, the prevalence of non-RS has been shown to be reduced (and RS elevated) with increasing age [18, 19]; that is consistent with our present results, indicating a significant increase of RS with age. We found RS to be associated with many factors, such as exercise, alcohol consumption, and smoking, which is also consistent with previous studies [18, 19]. Because exercise, alcohol consumption, and smoking have been associated with the quality and quantity of sleep [21, 22], the RS examined in this study probably reflects aspects of satisfactory sleep. Given that RS is likely to be reported after good sleep, our previous finding of a high prevalence of longer sleep duration in individuals with SHL [13] may not have reflected poor sleep quality.

Our previous study suggested that SHL is associated with longer sleep duration. In a longitudinal study, the risk of longer sleep duration (≥ 8 h) after the age of 8 years was significantly greater in subjects with SHL at 4000 Hz at baseline. However, it is not known whether SHL could be a cause of RS; that and other unquantified variables could confound the associations identified in the present study. Although RS itself may be good for general health, it could also reflect certain conditions that link to early hearing loss. In recent years, ARHL has been reportedly associated with cognitive impairment and dementia [23, 24]; those factors may also be related to longer sleep duration [25–27]. The auditory system involves both the ear (peripheral) and brain (central) [28]. Taken together, these findings indicate that early hearing impairment may be associated with RS or longer sleep duration through the etiology of cognitive impairment and dementia; however, in our main analysis, we excluded older subjects aged 70–79 years. Intriguingly, we found that the proportion of RS was not highest in subjects with bilateral SHL at 4000 Hz in the oldest group, aged 70–79 years (Additional file 2: Figure S1). One plausible explanation is that the association between SHL and RS may be modified by age-related insomnia [29, 30] and common complications, such as urinary tract symptoms [16, 31].

Regular noise exposure may confound the current findings because noise-induced hearing loss begins at around 4000 Hz [32–35]. Unfortunately, hearing at frequencies beyond 4000 Hz and tinnitus were not investigated in the present study; thus, we could not differentiate between noise-induced hearing loss and ARHL. Noise exposure in the daytime elicits noise-induced hearing loss; it may increase the requirement for longer sleep duration or RS owing to the listening effort required and cognitive fatigue induced [36–39].

In conclusion, our present and previous studies suggest an unexpected potential link between early hearing impairment and good sleep; they may be preliminary observations for future multidisciplinary work. Additional larger studies are required to confirm our results and investigate the underlying mechanisms and true clinical relevance.

## Limitations

This was a cross-sectional study and did not allow us to conclude the causality between RS and SHL.

Detailed assessment of sleep quality, for example, by polysomnography and a specialized questionnaire as in previous studies [40, 41], and of hearing loss (particularly including other frequencies) is required to elucidate the features of RS and impaired hearing.

Investigation of brain dysfunctions, such as cognitive impairment and dementia, may also be required.

## Additional files

**Additional file 1: Figure S1.** Proportion of subjects with RS categorized every 5 years. The small vertical bars represent the standard error with RS numbered as 1 and non-RS as 0. The proportion of subjects with RS significantly rose with increasing SHL in subjects aged 60–64 years (*P* = 0.001, one-way ANOVA). *RS* restorative sleep.

**Additional file 2: Figure S1.** Average age. The small vertical bars represent the standard error. The average age significantly rose with increasing SHL in all age-groups (all *P* < 0.0001, one-way ANOVA). *RS* restorative sleep.

**Additional file 3: Figure S1.** Proportion of subjects with RS aged 20–79 years. The small vertical bars represent the standard error with RS numbered as 1 and non-RS as 0. The statistical results and numbers of subjects aged 40–69 years are the same as in Fig. 1 and not indicated. The data of SHL were unavailable in 165 subjects aged 70–79 years. RS, restorative sleep.

## Abbreviations

ANOVA: analysis of variance
ARHL: age-related hearing loss
OR (95%CI): odds ratio (95% confidence intervals)
RS: restorative sleep
SHL: subclinical hearing loss
