# Supplementary material for: Unexpected association between subclinical hearing loss and restorative sleep in a middle-aged and elderly Japanese population
Source: BMC Res Notes. 2018 Mar 27;11:195. doi: 10.1186/s13104-018-3315-8 (PMC5870928; doi:10.1186/s13104-018-3315-8)
Supplement: Supplementary file 1 — Additional file 1: Figure S1. Proportion of subjects with RS categorized every 5 years. The small vertical bars represent the standard error with RS numbered as 1 and non-RS as 0. The proportion of subjects with RS significantly rose with increasing SHL in subjects aged 60–64 years (P = 0.001, one-way ANOVA). RS restorative sleep. [file 13104_2018_3315_MOESM1_ESM.docx]

40

50

60

70

80

40 - 44

45 - 49

50 - 54

55 - 59

60 - 64

65 - 69

Bilateral SHL

Unilateral SHL

Intact hearing

4000 Hz

*p* = 0.01

Proportions of subjects with RS (%)

Age (years old)
