# Supplementary material for: Unexpected association between subclinical hearing loss and restorative sleep in a middle-aged and elderly Japanese population
Source: BMC Res Notes. 2018 Mar 27;11:195. doi: 10.1186/s13104-018-3315-8 (PMC5870928; doi:10.1186/s13104-018-3315-8)
Supplement: Supplementary file 2 — Additional file 2: Figure S1. Average age. The small vertical bars represent the standard error. The average age significantly rose with increasing SHL in all age-groups (all P < 0.0001, one-way ANOVA). RS restorative sleep. [file 13104_2018_3315_MOESM2_ESM.docx]

30

35

40

45

50

55

60

65

70

40 - 44

45 - 49

50 - 54

55 - 59

60 - 64

65 - 69

Bilateral SHL

Unilateral SHL

Intact hearing

Averages of age (years)

Age (years old)
