# Supplementary material for: Unexpected association between subclinical hearing loss and restorative sleep in a middle-aged and elderly Japanese population
Source: BMC Res Notes. 2018 Mar 27;11:195. doi: 10.1186/s13104-018-3315-8 (PMC5870928; doi:10.1186/s13104-018-3315-8)
Supplement: Supplementary file 3 — Additional file 3: Figure S1. Proportion of subjects with RS aged 20–79 years. The small vertical bars represent the standard error with RS numbered as 1 and non-RS as 0. The statistical results and numbers of subjects aged 40–69 years are the same as in Fig. 1 and not indicated. The data of SHL were unavailable in 165 subjects aged 70–79 years. RS, restorative sleep. [file 13104_2018_3315_MOESM3_ESM.docx]

40

50

60

70

80

90

40 - 49

50 - 59

60 - 69

Bilateral SHL

Unilateral SHL

Intact hearing

30 - 39

20 - 29

70 - 79

Age (years old)

12,172

64

15,033

241

90

17

212

94

206

4000 Hz

Proportions of subjects with RS (%)
